# The Complex World of Regulation of Pituitary Growth Hormone Secretion: The Role of Ghrelin, Klotho, and Nesfatins in It

**DOI:** 10.3389/fendo.2021.636403

**Published:** 2021-03-11

**Authors:** Jesús Devesa

**Affiliations:** Scientific and Medical Direction, Medical Center Foltra, Teo, Spain

**Keywords:** growth hormone, IGF-I, GHRH, somatostatin, ghrelin, klotho, nesfatins

## Abstract

The classic concept of how pituitary GH is regulated by somatostatin and GHRH has changed in recent years, following the discovery of peripheral hormones involved in the regulation of energy homeostasis and mineral homeostasis. These hormones are ghrelin, nesfatins, and klotho. Ghrelin is an orexigenic hormone, released primarily by the gastric mucosa, although it is widely expressed in many different tissues, including the central nervous system and the pituitary. To be active, ghrelin must bind to an n-octanoyl group (n = 8, generally) on serine 3, forming acyl ghrelin which can then bind and activate a G-protein-coupled receptor leading to phospholipase C activation that induces the formation of inositol 1,4,5-triphosphate and diacylglycerol that produce an increase in cytosolic calcium that allows the release of GH. In addition to its direct action on somatotrophs, ghrelin co-localizes with GHRH in several neurons, facilitating its release by inhibiting somatostatin, and acts synergistically with GHRH stimulating the synthesis and secretion of pituitary GH. Gastric ghrelin production declines with age, as does GH. Klotho is an anti-aging agent, produced mainly in the kidneys, whose soluble circulating form directly induces GH secretion through the activation of ERK1/2 and inhibits the inhibitory effect that IGF-I exerts on GH. Children and adults with untreated GH-deficiency show reduced plasma levels of klotho, but treatment with GH restores them to normal values. Deletions or mutations of the *Klotho* gene affect GH production. Nesfatins 1 and 2 are satiety hormones, they inhibit food intake. They have been found in GH3 cell cultures where they significantly reduce the expression of *gh* mRNA and that of pituitary-specific positive transcription factor 1, consequently acting as inhibitors of GH production. This is a consequence of the down-regulation of the cAMP/PKA/CREB signaling pathway. Interestingly, nesfatins eliminate the strong positive effect that ghrelin has on GH synthesis and secretion. Throughout this review, we will attempt to broadly analyze the role of these hormones in the complex world of GH regulation, a world in which these hormones already play a very important role.

## Introduction

Our group was the first to describe in humans the existence of an intrinsichypothalamic-somatotrophic rhythm, sexually dimorphic, that conditions the secretion of growth hormone (GH) (1). We also described for the first time that this rhythm depends mainly on the negative tonic effect of somatostatin (SS) on hypothalamic GHRH release and pituitary GH secretion (2). Several studies by our group showed that the control of SS and GHRH depends on the rate of delivery of hypothalamic norepinephrine (NA) to the SS and GHRH neurons (3–7). The role of catecholamines in the control of GH release is related to the classically known metabolic actions of GH as a counterregulatory (hyperglycemic-inducing), lipolytic, and anabolic hormone. This is the reason why most of the tests used to analyze deficient GH secretion are based on increasing the supply of NA to SS neurons, as occurs with the administration of insulin (ITT) to induce hypoglycemia and consequently the release of NA, or the administration of clonidine, an alpha 2 agonist, alone or followed by the administration of GHRH (8). Other classical tests, such as arginine administration, are known to inhibit SS secretion (9, 10), but the exact mechanism by which it occurs has not yet been established. However, in recent years, the complex world of GH secretion regulation has changed due to two main factors:

1. We currently know that GH is a pleiotropic hormone that, in addition to its metabolic and growth effects, has very important positive effects on practically all organs and tissues (11).
2. GH expression has been shown to exist at many extra-pituitary sites, including the nervous system, reproductive system, immune system, cardiovascular system, muscle tissue, dermal tissue, skeletal tissue, and even in the eyes (11–14), where the hormone exerts physiological or pathological auto/paracrine roles.

It seems logical, then, that both the knowledge of the multiple functions that GH plays in the body and the peripheral expression of the hormone have increased the knowledge of the complexity of GH regulation, far beyond the classical concept (2), with the discovery of new factors involved in neuroregulation and/or paracrine regulation of the expression of this hormone. In this review, we will analyze how three of these factors, ghrelin, klotho, and nesfatins, act on the expression and release of pituitary GH.

## Ghrelin

**Figure 1:**
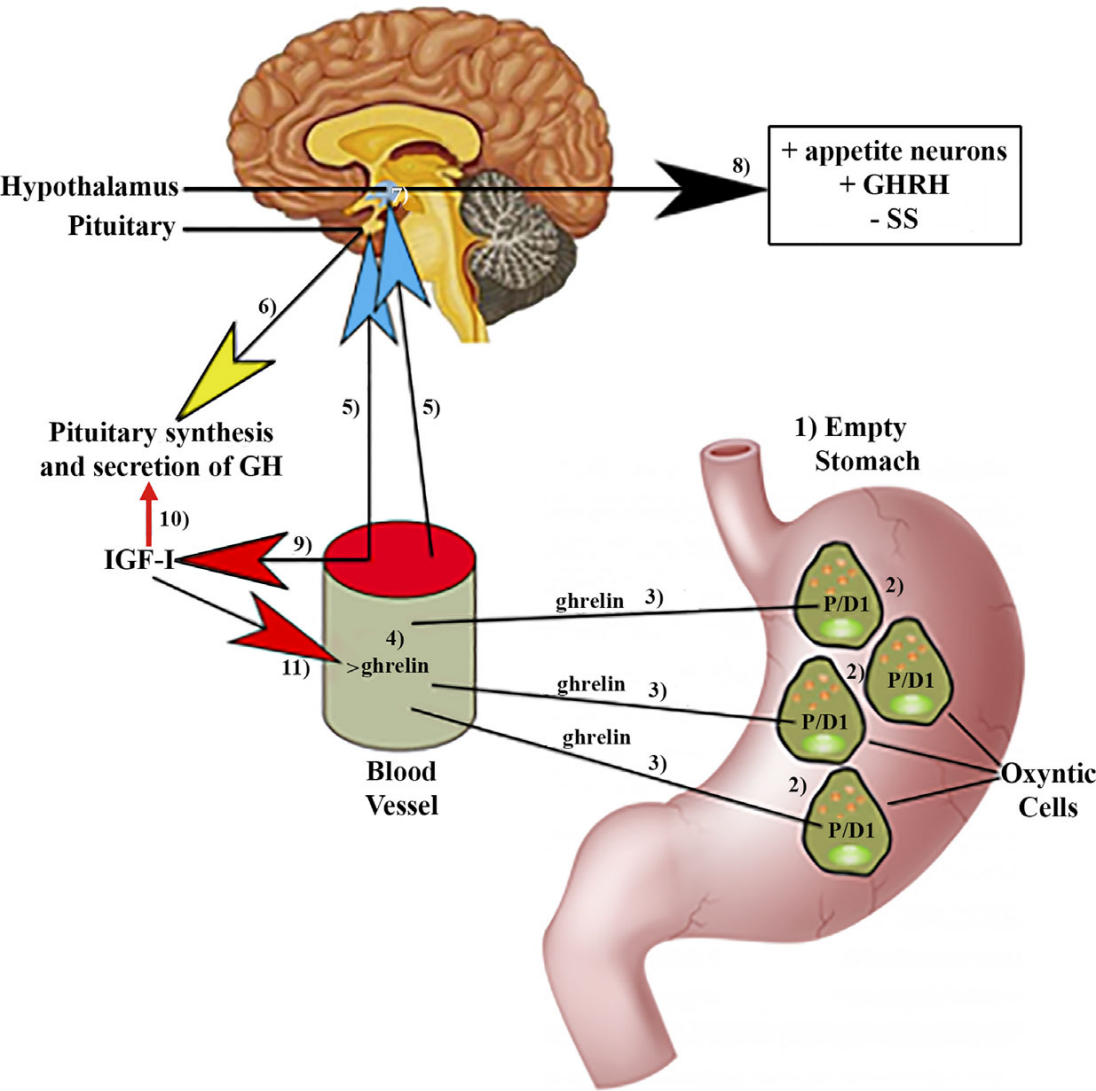
Ghrelin production by the stomach and effects of ghrelin on pituitary GH synthesis and secretion. (1)In fasting situations, an empty stomach increases ghrelin production in specific P/DI cells (2) in humans located in oxyntic cells. Then, ghrelin is released into the circulation (3, 4) from where it reaches (5) the pituitary gland, inducing the synthesis and secretion of GH (6). Circulating ghrelin also reaches the hypothalamus (7), where it induces stimulation (8) of the appetite neurons, GHRH, and inhibits the release of SS. (9)Furthermore, circulating ghrelin inhibits the inhibitory effects of IGF-I on the synthesis and pituitary secretion of GH (10). In turn, IGF-I inhibits the increase in circulating ghrelin (11) produced in response to fasting. Blue arrows: stimulation; Red arrows: Inhibition; Yellow arrow: synthesis and secretion of GH; Black arrow: Hypothalamic effects of ghrelin; +, stimulation; −, inhibition.

In 1990 it was published that a synthetic hexapeptide called GH-releasing peptide (GHRP) could act as a potent GH secretagogue in normal humans (15). Furthermore, the effect of this peptide was independent from that of GHRH and it acted synergistically with this GH-releasing hormone discovered a few years earlier (16, 17). The discovery of synthetic GH secretagogues (GHS) led to investigate on how they could act on the secretion of this hormone. Thus, in 1996 a heterotrimeric receptor coupled to a GTP-binding protein was discovered; it was present in the pituitary and arcuate ventromedial and infundibular hypothalamus of several species, including humans (18). Detection of such a receptor implied that an unknown natural hormone had to exist. This hormone was identified and purified in the rat stomach in 1999 and was called ghrelin (19). Ghrelin is a 28 amino acid peptide hormone in which the third amino acid, normally serine, is modified by a fatty acid, a key modification for ghrelin activity (20). Therefore, GH secretion from the pituitary is regulated not only by the GHRH-SS interaction, but also by gastric ghrelin. The question should now be: why does a gastric hormone play a positive role in GH secretion? The answer to this question is given by the different actions that ghrelin performs in the body. Soon after its characterization, it was found that ghrelin is an orexigenic hormone that is present in the blood in times of fasting and reaches the central nervous system to which it transmits a hunger signal. This is the reason why patients with anorexia nervosa normally show increased plasma concentrations of ghrelin, whereas in obesity they are reduced (21). These facts also explain why GH secretion is increased in anorexia nervosa and reduced in obesity, and also why there is an age-related decrease in plasma ghrelin concentrations in the elderly, a stage of life in which GH secretion is practically absent and there is a decrease in appetite (21). Based on these apparently unrelated effects of ghrelin, the stimulation of hunger and the induction of GH secretion in the pituitary, it is feasible to think that ghrelin appeared in evolution to induce eating behavior and optimize the use of food digested by promoting the release of an anabolic hormone, as GH (22). These concepts are schematized in **Figure 1**.

**Figure 2:**
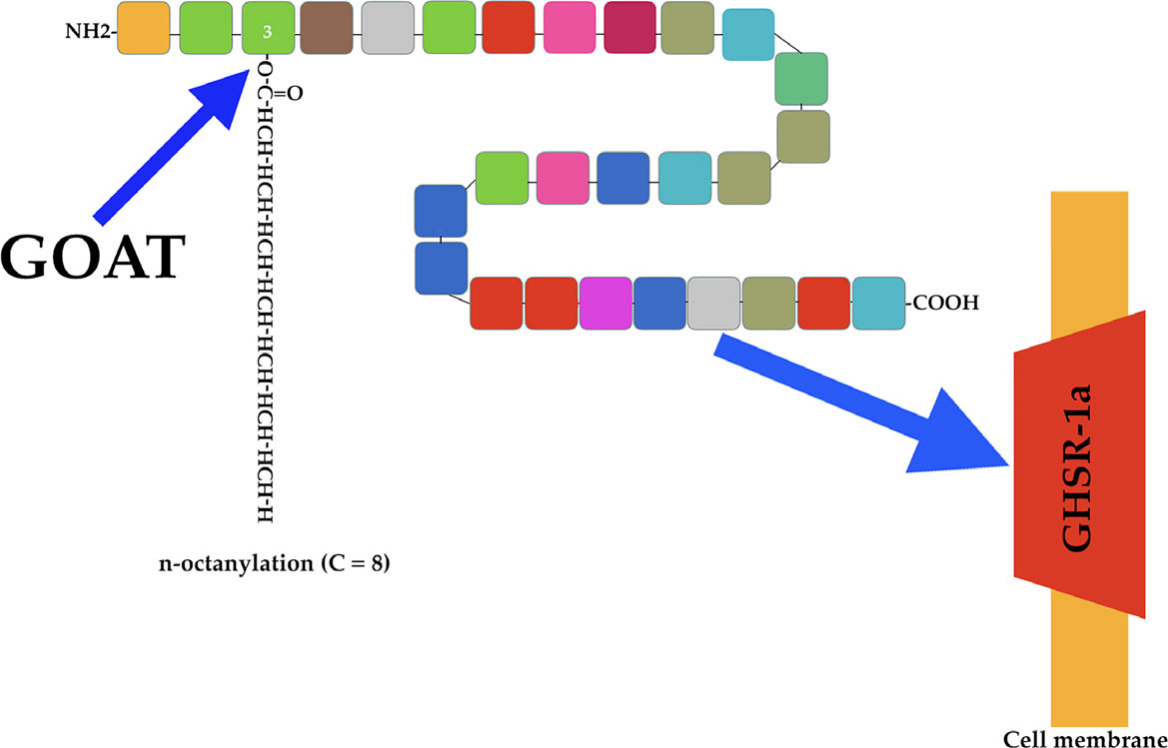
Ghrelin acylation and ghrelin receptor. The endocrine active form of ghrelin is produced by attaching an n-octanoyl group (usually 8 C) to serine at position 3 of the ghrelin molecule (blue arrow). This is done by the enzyme ghrelin-O-acyl-transferase (GOAT). Active acyl ghrelin acts on a receptor (blue arrow) expressed mainly in the pituitary and hypothalamus. This receptor is a G-protein-coupled receptor (GHSR-1a), and cannot be activated by des-acyl ghrelin.

There are two forms of ghrelin: acyl ghrelin (octanoylated form) and des-acyl ghrelin (non-octanoylated form). The former is the active form responsible for most of the physiological functions of this peptide, including the induction of pituitary GH secretion. Acyl ghrelin is produced by attaching an n-octanoyl group to serine at position 3 (23) (**Figure 2**).

As indicated above, the discovery of ghrelin occurred as a consequence of the interest in the search for a natural hormone capable of interacting with the receptor for synthetic secretagogues that induce GH secretion. This receptor is a G-protein-coupled receptor (GHSR-1a) expressed mainly in the pituitary and hypothalamus, and responsible for mediating the endocrine activities of acyl ghrelin (**Figure 2**). This receptor cannot be activated by des-acyl ghrelin (19, 24, 25), which despite being more abundant than acyl ghrelin lacks known endocrine activity (26), although more studies are needed to better understanding its actions.

### *Ghrelin* Gene and Ghrelin Production Sites

The *Ghrelin* gene is made up of six exons and three introns, located on chromosome 3, at the 3p25-2 locus, although the first exon made up of 20 bp is a non-coding exon (exon 0 of 20 bp) (27). In humans, ghrelin is produced primarily in P/D1 cells (X/A-like cells in rats) and is distributed throughout the stomach mucosa (28, 29); the greatest amount of ghrelin found in plasma comes from these cells (30, 31) (**Figure 1**).

However, despite the fact that after its discovery, ghrelin was considered a “hunger hormone” and a regulator of GH secretion in the pituitary, current data indicate that it is a pleiotropic hormone that exerts many different effects on the human body and, therefore, can be produced, as its receptor, in a wide variety of tissues and organs. Besides the stomach, ghrelin and its receptor are expressed in many different regions of the brain (32–34), pituitary (35, 36), kidneys (34), heart (37, 38), lung (34), ovaries (34), intestine (39), and pancreatic islets (40, 41). This indicates that the hormone exerts multiple actions, endocrine and/or auto/paracrine, in these tissues, although this is not the objective of this review.

### Ghrelin Acylation and Secretion

As stated above, ghrelin requires acylation to interact with and activate its receptor. This acylation is performed by ghrelin O-acyl-transferase (GOAT), which links a fatty acid side chain (C8) to serine 3 of ghrelin (42, 43) (**Figure 2**). At this point is of interest to highlight that the lipids involved in this acylation are mainly those present in nutrition because the ghrelin-producing cells in the stomach are located within the oxyntic gastric glands, which allows direct access to the ingested lipids (44), mainly middle chain fatty acids, because they can be absorbed into the circulation without undergoing breakdown by lipases and bile acids (45).

The GOAT-ghrelin system appears to be a nutrient sensor to signal to the brain that calorie-rich foods are available, leading to optimization of nutrient partitioning and growth signals (46, 47).

Acyl ghrelin is deacylated by plasma esterases and then degraded by plasma proteases and excreted in the urine.

Knowledge about how gastric ghrelin secretion is regulated can be useful to know how this peptide acts on GH secretion in the pituitary.

**Figure 3:**
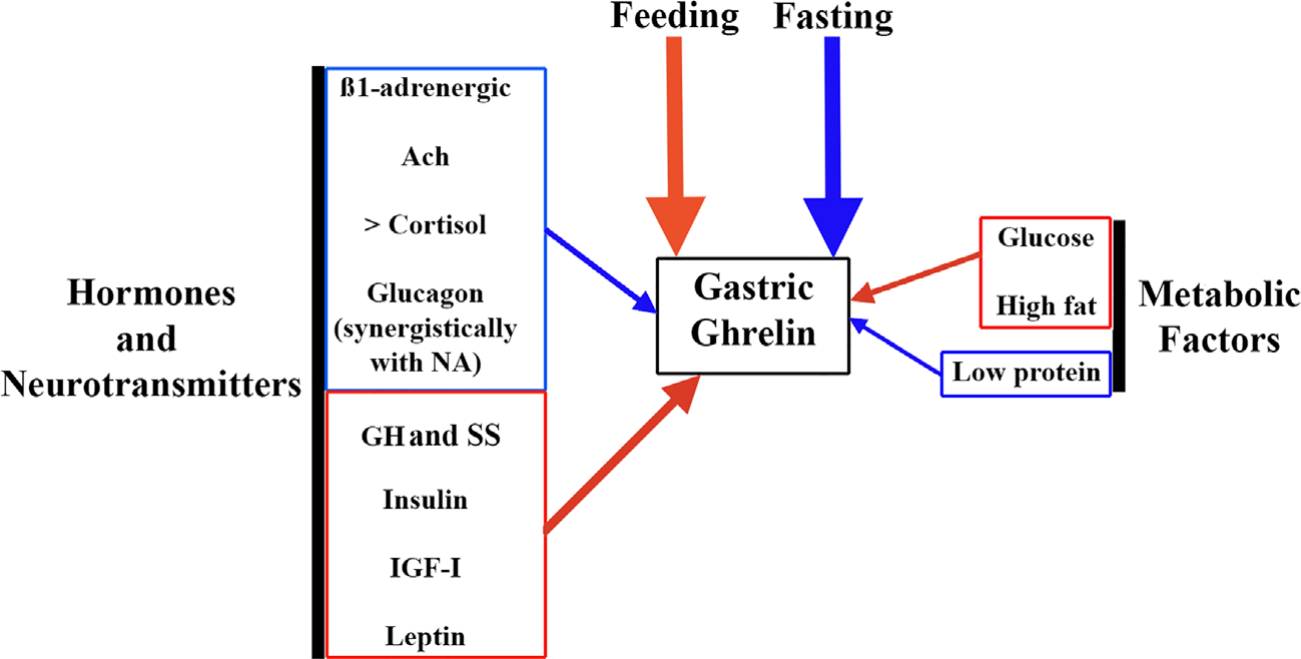
Factors involved in the regulation of gastric ghrelin secretion. As ghrelin is an orexigenic hormone, the main factors that regulate its gastric secretion are fasting (stimulates it, blue arrow) and feeding (inhibits it, red arrow). But in addition, a series of factors such as hormones and neurotransmitters, and metabolic factors play an important role in this regulation. Among neurotransmitters, ß-1 adrenergic pathways and the cholinergic system (Ach) stimulate gastric ghrelin production (blue arrow). Among the hormones, increased plasma cortisol and glucagon levels also stimulate gastric ghrelin production, although in the case of glucagon its effect is most likely to depend on an action carried out synergistically with NA. In contrast, other hormones such as GH, SS, insulin, IGF-I and leptin inhibit gastric ghrelin production (red arrow). Among metabolic factors, glucose and high fat content inhibit gastric ghrelin production (red arrow), while low protein intake stimulates it (blue arrow).

Gastric ghrelin synthesis and secretion increase during fasting and decrease during feeding (48). This is the reason why chronic intake of high-calorie diets, prolonged ingestion of high fats, and obesity lead to a reduction in gastric ghrelin production and secretion (48, 49), while a low protein supply significantly increases plasma ghrelin (49) (**Figure 3**).

Interestingly, some studies have shown that ghrelin secretion increases in response to stimulation of the sympathetic nerves (50, 51) or by local infusion of adrenergic hormones in the stomach (52), while SS inhibits it (52).

Adrenergic hormones stimulate the release of gastric ghrelin by acting directly on the ß1 receptors of ghrelin-producing cells, especially rich in this type of adrenergic receptors (53). In the same study, the authors confirmed the role of these ß1 receptors in ghrelin production by administering the ß1 receptor blocker atenolol. This prevented the increase in plasma ghrelin that appears after fasting. Furthermore, when they depleted neuronal catecholamines with reserpine, they again observed that there was no ghrelin release after fasting. All this led these authors to propose that fasting acts on gastric ghrelin-secreting cells through the sympathetic nervous system (53). Of course, alpha-adrenergic antagonists also induce an increase in plasma ghrelin concentration (30), as does the administration of muscarinic agonists (54). Furthermore, excitation of the vagus nerve in the gastric mucosa directly stimulates ghrelin-producing cells (55).

As indicated in the *Introduction*, the autonomic nervous system plays a prominent role in the neuroregulation of GH (2), but also in the regulation of gastric ghrelin secretion, as we have just seen. What then are the relationships between these two hormones?

In addition to the autonomic system, some hormones and metabolic factors contribute to modulate gastric ghrelin production. For example, GH exerts a negative feedback effect on ghrelin production and secretion, which makes sense (56). More complex is the relationship between insulin and gastric ghrelin. Insulin has been reported to affect ghrelin production and signaling (57), but the reverse is also true (58). The mammalian target of rapamycine (mTOR) is closely involved in metabolic changes in various tissues after postprandial insulin secretion (59), and plays a key role in insulin signaling (60). Components of the mTOR signaling pathway are expressed in endocrine cells of the gastric mucosa, and most of these ghrelin-producing cells show positivity when stained for these components of the mTOR signaling pathway (61). On the contrary, physiological levels of ghrelin impair the functions of pancreatic ß-cells, inhibiting insulin secretion of (62). Most likely, this inhibitory effect depends on the stimulation of pancreatic SS production (63). The intricate relationships between ghrelin and insulin, both peripherally and in the CNS, deserve further explanation, but this is beyond the scope of this review. In any case, it is clear that both hormones play an important role in balancing energy expenditure and metabolic homeostasis.

Another important hormone, such as cortisol, exerts a positive effect on gastric ghrelin secretion (64), which seems to depend directly on cortisol itself and not on CRH or ACTH, since although plasma ghrelin concentrations increase in response to stimulation by ACTH (induced by stress or after exogenous ACTH administration), when metyrapone (which blocks cortisol synthesis) was administered, ACTH increases but plasma ghrelin levels decrease (64). This and other studies by the same group indicate that the hypothalamic-pituitary-adrenal axis is only capable of increasing ghrelin secretion when plasma cortisol is elevated (64). Furthermore, this positive effect of cortisol seems to depend on its plasma levels.

In the case of glucagon, another hormone related to metabolism, mainly glucose homeostasis, this hormone has been shown to induce a significant decrease in ghrelin secretion, which seems not to depend on changes in glucose or insulin concentrations (65), nor in ghrelin-producing cells in the stomach (66), and is suppressed when there is a lesion in the hypothalamic-pituitary axis (65), suggesting that the inhibitory effect of glucagon on ghrelin release is exerted at the hypothalamic-pituitary level, perhaps inducing hypothalamic somatostatin release (67). However, another study indicates that glucagon may participate in the pre-prandial peak of ghrelin, because: a) the glucagon receptor exists in the endocrine cells of the gastric mucosa; b) ghrelin increases in rat plasma during glucagon perfusion; c) glucagon can stimulate ghrelin gene transcription. These led to the claim that ghrelin can be directly regulated by glucagon which acts synergistically with NA (68).

Leptin is another hormone involved in controlling ghrelin release, which is logical given its effects, different from ghrelin, on appetite control. Leptin significantly reduces plasma ghrelin levels and decreases food intake. The weight-reducing effects of leptin are exerted by its direct central effects on the hypothalamus and by its inhibitory actions on gastric release and central actions of ghrelin (69).

Since plasma levels of IGF-I are important mediators of most of the peripheral actions of GH, it is reasonable to assume that there must be important relationships between IGF-I and gastric ghrelin secretion. Data obtained from a large cohort of middle-aged subjects indicate that plasma IGF-I concentration is a significant determinant of plasma ghrelin concentration, with a negative correlation between them (70). A similar negative correlation had previously been found in children and adolescents (71–73). It is important to note that a large amount of circulating IGF-I binds to the transporter protein IGFBP3, so when analyzing the relationships between IGF-I and ghrelin, only free IGF-I, which is the bioactive form, should be considered. On this basis, the highest concentration of ghrelin was observed in GH-deficient children in whom there was low bioavailability of IGF-I (74). That is, low plasma levels of IGF-I induce the synthesis and secretion of ghrelin, while in turn ghrelin decreases plasma levels of IGF-I.

Although many other hormones can help regulate gastric ghrelin synthesis and secretion, their role is not as relevant as that of the hormones just described.

With regard to metabolic factors, it is well known that plasma ghrelin concentrations decrease in normal subjects after oral or intravenous glucose administration (75). The effect of these factors on gastric ghrelin secretion is schematized in **Figure 3** and **Table 1**.

**Table 1:** Main factors involved in the regulation of the gastric secretion of ghrelin.

Factors	Inductors	Inhibitors
Hormones and neurotransmitters	ß1-adrenergic stimulation Alpha-adrenergic antagonism	GH and Somatostatin
	Ach	Insulin
	> Plasma cortisol	Plasma IGF-I
	Glucagon	Leptin
Food	Fasting	Feeding
Metabolic Factors	Low protein intake	Glucose, High fat

Different factors can act on the gastric secretion of ghrelin, either stimulating it or inhibiting its secretion. >, Increased.

To finish this subsection on how gastric ghrelin synthesis and secretion is regulated, it seems essential to describe a recent study in which it is reported that there are specific cellular mechanisms for the detection of nutrients in the mouse stomach; these are chemosensors expressed primarily in a region-specific way in gastric and stomach ghrelin cells, and their function is to modulate gastrointestinal responses to food intake, for example by inhibiting ghrelin secretion (76).

### Ghrelin and Pituitary GH Secretion

**Figure 4:**
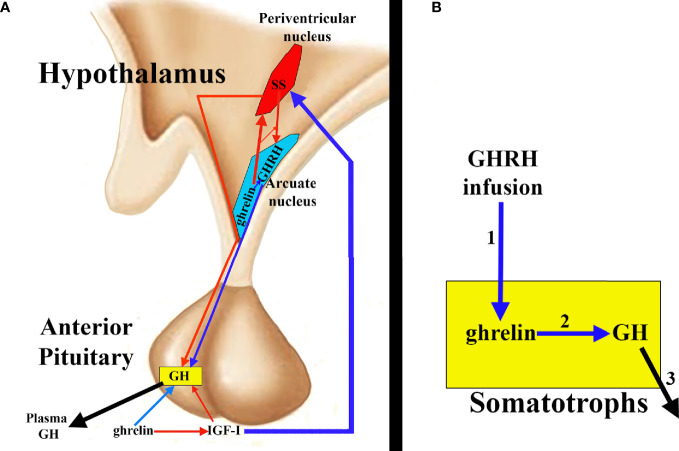
Ghrelin and pituitary GH secretion. **(A)** Ghrelin directly stimulates pituitary GH secretion (blue arrow), but indirectly it also contributes to this secretion, since ghrelin inhibits IGF-I (red arrow) and, therefore, the inhibitory effect of this peptide on GH; furthermore, the inhibition of IGF-I impedes the stimulatory effect of this hormone on the hypothalamic release of SS (blue arrow), which allows the release of GHRH to the portal circulation (blue arrow) with its consequent positive effect on the synthesis and secretion of GH. Furthermore, it appears that ghrelin co-localizes with GHRH in the hypothalamic arcuate nucleus, inducing its release, directly or inhibiting the release of SS from the periventricular nucleus (red arrow), thus inhibiting the inhibitory effect that SS exerts on the release of GHRH and on the synthesis and secretion of pituitary GH. **(B)** ghrelin and its receptor are expressed in the pituitary, where they could play an auto/paracrine role in the regulation of GH release. In fact, GHRH infusion (1) increases pituitary ghrelin mRNA levels that could induce GH stimulation (2) and release (3).

Ghrelin stimulates GH release in the pituitary by acting at two levels: 1) directly on the pituitary somatotrophs and 2) antagonizing the hypothalamic and pituitary effects of SS and inducing GHRH secretion. Indirectly, a third mechanism of action could be considered since ghrelin decreases plasma levels of IGF-I, consequently inhibiting the negative effect of IGF-I on GH secretion exerted directly on the somatotrophs, and indirectly because IGF-I induces hypothalamic secretion of SS (2) (**Figure 4A**).

The first clear evidence that in addition to GHRH and SS, another factor had to be involved in the regulation of GH secretion came from several studies in humans in which it was shown that the nocturnal increase in GH was not inhibited by the infusion of octreotide, an analog of SS (77, 78). The last of these studies proposed that ghrelin could be responsible for the diurnal rhythm of GH secretion (78). This confirms other studies in which it was shown that patients with inactive GHRH receptor, due to mutations, still had rhythmic GH secretion, suggesting that another factor, in addition to GHRH, was acting on pituitary GH secretion (79).

There is a possibility that ghrelin interacts with GHRH at the hypothalamic level, since it has been shown that transgenic rats that have a decreased expression of the ghrelin receptor GHSR-1a in the arcuate nucleus of the hypothalamus, where GHRH is produced, show a decrease of GHRH in the neurons that produce it (80). Administration of a ghrelin receptor antagonist leads to a decrease in the amplitude of GH pulses in rodents (81). Consistent with this finding, in humans, a non-sense mutation affecting ghrelin receptor activity is associated with short-stature (82). This possibility of a hypothalamic ghrelin-GHRH interaction appears to be reinforced by the finding of ghrelin in the hypothalamic arcuate nucleus from where it increases GHRH release and antagonizes the inhibitory effects of SS (83) (**Figure 4A**).

The complicated relationships between ghrelin, GHRH, and SS in controlling episodic GH release were extensively analyzed in an elegant study conducted in male rats in 2003 (84). In that study, intravenous administration of ghrelin during a physiological GH peak was shown to induce a marked increase in plasma GH, suggesting that ghrelin acted synergistically with GHRH; this required the integrity of a functional GHRH system, because the immunoneutralization of GHRH led to a virtual absence of ghrelin-induced GH secretion; however, when ghrelin was administered during a physiological trough period the GH response was clearly attenuated, although a recovery in its secretion was observed 15 min after ghrelin administration. Immunoneutralization of SS reversed the early blunted response to ghrelin in the trough periods, the GH response being similar to that observed when ghrelin was administered during episodes of peak GH. This indicates that ghrelin is a functional SS antagonist. Interestingly, when ghrelin was administered *via* intracerebroventricular during a trough period, no increase in plasma GH was observed, indicating that SS may behave as a functional ghrelin antagonist that acts centrally (in GHRH neurons) and in the pituitary gland (84).

As noted above, ghrelin and its receptor are also expressed in the pituitary (35, 36), thus the possibility exists that pituitary ghrelin plays an auto/paracrine role in the regulation of GH release. GHRH infusion increases pituitary ghrelin mRNA levels, suggesting that GHRH may be a regulator of pituitary ghrelin production (85) (**Figure 4B**). In situations where GHRH expression increases (GH deficiency due to GH gene mutations, hypothyroidism, etc.), pituitary ghrelin expression also increases; conversely, when hypothalamic GHRH expression decreases (GH replacement therapies, glucocorticoid treatments, hyperthyroidism, etc.), pituitary ghrelin expression also decreases. All of this suggests that pituitary ghrelin is dependent on an adequate supply of GHRH to the pituitary gland. GHRH stimulation of pituitary cell cultures in the presence of a specific ghrelin receptor inhibitor significantly decreased the GH response to GHRH challenge, although this effect was not observed in the absence of GHRH stimulation. These results suggest that pituitary ghrelin may act physiologically on GH secretion, improving or optimizing the response of somatotrophs to GHRH. **Table 2** summarizes these effects of ghrelin on pituitary GH secretion.

**Table 2:** Ghrelin and pituitary GH secretion.

Level	Effect
Anterior Pituitary	Direct effect on GH secretion by inhibiting SS action
Arcuate nucleus Periventricular nucleus	Increases GHRH releaseAntagonizes the inhibitory effects of SS on GHRH release
Anterior Pituitary	Inhibits the inhibitory effect of IGF-I on GH secretion
Anterior Pituitary	Acts synergistically with GHRH in inducing GH secretion
Anterior Pituitary	Auto/paracrine induction of GH secretion dependent on GHRH supply.
Anterior Pituitary	> Transcription Na+ channels> NOS/NO+ PLC > > Ca2+

Effects of ghrelin on GH secretion and levels at which it acts. >, Increases. +, Stimulates.

**Figure 5:**
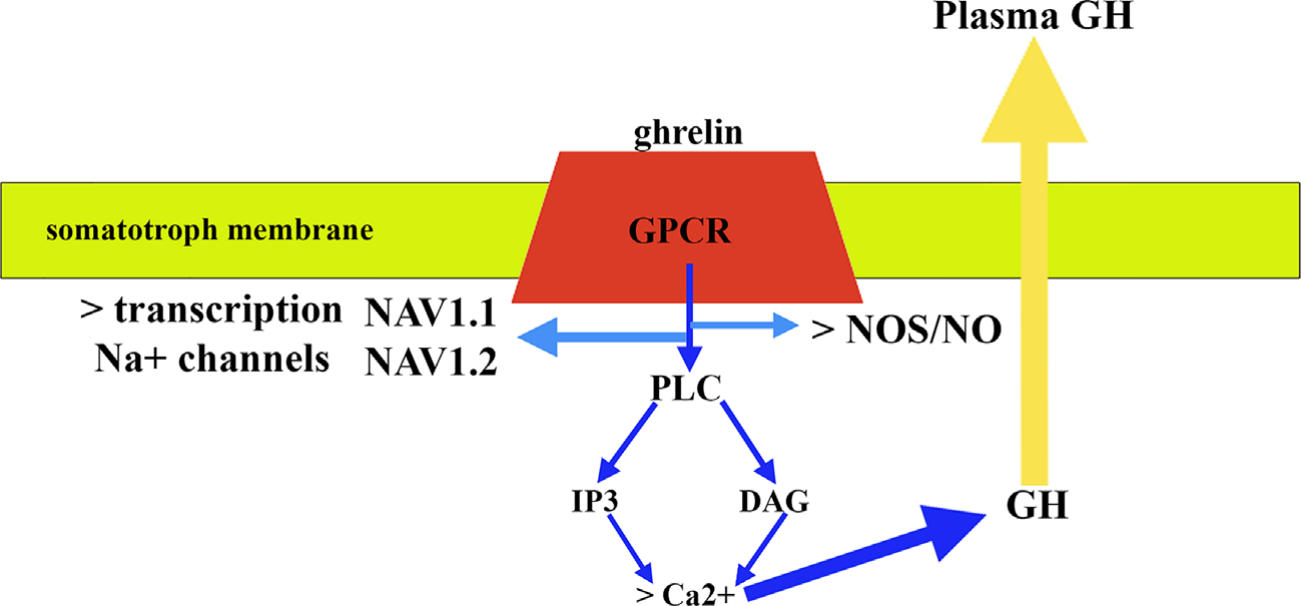
Mechanism of action of ghrelin in pituitary somatotrophs. After binding to a G-protein-coupled receptor (GPR) and activating it, the signaling pathways involve stimulation of phospholipase C (PLC) activity. This activation leads to the formation of inositol 1,4,5 triphosphate (IP3) and diacylglycerol (DAG) (blue arrows). Both IP3 and DAG induce an increase in cytosolic calcium that allows GH release (yellow arrow), although activation of NOS/NO pathway is also required. In addition, the effect of ghrelin includes the activation of the transcription of the Na+ channel gene that leads to the expression of two isoforms of Na+: NAV1.1 and NAV1.2.

Regarding the signaling pathways involved in the release of GH, GHRH binds to a G-protein-coupled receptor (GPCR) that once activated induces the activation of adenylyl cyclase that generates the conversion of ATP to cAMP; cAMP induces a conformational change in protein kinase A (PKA) regulatory subunits that allows phosphorylation of serine or threonine residues in proteins that are then activated (83). Ghrelin also acts through a GPCR but in this case the activation of this receptor leads to the stimulation of the activity of phospholipase C (PLC) that induces the formation of inositol 1,4,5-triphosphate (IP3), and diacylglycerol (DAG) (**Figure 5**); both IP3 and DAG induce an increase in cytosolic calcium that allows the release of GH (83). Furthermore, *in vitro* studies demonstrated that ghrelin requires activation of the NOS/NO pathway and its subsequent guanylate cyclase (GC)/cGMP signal transduction pathway to induce GH release from the pituitary (86). A more recent study demonstrated that GH release from cultured bovine somatotrophs during chronic ghrelin treatment is associated with a significant increase in Na^+^ macroscopic current, the blockade of which with tetrodotoxin (TTX) nullifies GH release induced by ghrelin (87). In this study, it was also observed that chronic treatment with ghrelin produced an upregulation of GH transcription levels, as well as that of two isoforms of Na^+^ channels sensitive to TTX expressed in somatotrophs, such as NaV1.1 and NaV1.2, indicating that ghrelin also regulates the expression of the Na^+^ channel gene in these pituitary cells (87) (**Figure 5**). Of interest here is the recent description that AMP-activated protein kinase (AMPK), a hypothalamic enzymatic complex involved in the hypothalamic control of energy and metabolic homeostasis, and activated by ghrelin, participates in the control of GH secretion, as its blocking or its functional impairment inhibits ghrelin- or GHRH-induced GH secretion, most likely by increasing SS tone (88).

The great importance of ghrelin in the regulation of GH could perhaps be deduced from the data obtained in a study carried out in six healthy young male volunteers in which GH secretion was analyzed every 15 min (−30 to +210 min) in response to 1) iv administration of acyl ghrelin (1 µg/kg); 2) iv infusion of salbutamol (SLB; 0.06 µg/kg/min); 3) acyl ghrelin + SB; 4) saline infusion. While SB led to a significant inhibition of spontaneous GH secretion that remained abolished for up to 75 min after SB withdrawal, acyl ghrelin led to a marked increase in plasma GH levels that was unaffected by SB (89). Since SB is a ß-adrenergic agonist and ß-adrenergic agonists increase hypothalamic SS secretion and inhibit hypothalamic GHRH release (2), these data suggest that acyl ghrelin is refractory to the inhibitory effect of SS. Perhaps this is the mechanism by which gastric ghrelin acts at the pituitary level on the release of GH.

It is of interest now to analyze what happens to the secretion of ghrelin and GH in the elderly. It is well known that aging is associated with a decrease in GH secretion from the second decade of life (90). A similar age-related decline in circulating ghrelin levels has been reported (21, 91). However, the pituitary ghrelin receptor does not decline with aging, at least in mice (92), and the GH response to ghrelin is still seen in the elderly, although there is an age-related decline (93). This is an interesting study topic because, as described, senescence is associated with a decreased appetite (21). What is the reason why the secretion of an orexigenic hormone, such as ghrelin, and also that of an anabolic hormone, such as GH, is lost with aging?

### Summary

In summary ghrelin is a very complex hormone, because in addition to its two main actions: orexigenic and strong inducer of GH release in the pituitary, ghrelin exerts many different effects on practically the entire human body. The main source of ghrelin is the stomach, from where this hormone is released in response to different nervous, hormonal, and metabolic stimuli. These are related to the main action of ghrelin as a “hunger hormone,” which acts on the central nervous system to stimulate food intake. Additionally, ghrelin induces the secretion of pituitary GH, an anabolic hormone.

Ghrelin acts after being acylated by ghrelin O-acyl-transferase (GOAT), which binds a fatty acid side chain (C8) to serine 3 of ghrelin, forming acyl ghrelin, the active form, which acts on its receptor, a G-protein-coupled receptor (GHSR-1a).

The effects of acyl ghrelin on pituitary GH secretion occur both centrally and directly on pituitary somatotrophs. At the central level, ghrelin is expressed in many neurons that produce GHRH, which most likely facilitates the release of GHRH by antagonizing the inhibitory effect of somatostatin on this secretion. Furthermore, ghrelin exerts a synergistic effect with GHRH in the induction of GH secretion. At the pituitary level, acyl ghrelin has a direct effect on GH secretion; this effect probably depends on gastric ghrelin. Ghrelin expression has been detected in pituitary somatotrophs, which may suggest a paracrine effect of this peptide on GH secretion. Another effect of ghrelin on GH secretion comes from its inhibitory effects on plasma IGF-I. Since IGF-I inhibits GH secretion directly in somatotrophs and indirectly by activating hypothalamic somatostatin release, this action of ghrelin is another factor that positively contributes to GH secretion in the pituitary gland.

Finally, gastric ghrelin secretion decreases with age, as occurs with GH; however, the pituitary ghrelin receptor does not experience this decrease, and a GH response to ghrelin is still seen in the elderly.

## Klotho

Like ghrelin, the klotho transmembrane protein performs many different functions in the human body, among them it plays a very important role in controlling GH secretion. Klotho was first identified in 1997 as an anti-aging agent (94), since an impairment in its genetic expression, in mice, leads to a syndrome that mimics human aging: short lifespan, infertility, arteriosclerosis, skin atrophy, osteoporosis, and emphysema. Klotho-deficient mice (*kl/kl*) develop normally up to 3 weeks of age, but from this age they begin to show a severe aging phenotype, including growth retardation (94). Klotho was initially thought to be expressed in the distal tubules of the kidney and the choroid plexus of the brain, but was later shown in many different tissues, including the gonads and the pituitary gland. This may explain not only its role in the control of GH secretion, but also how it acts positively in pathological processes, such as arteriosclerosis, and many physiological processes in healthy humans (22). We now know that klotho is a circulating hormone that can be found in body fluids, including blood and cerebrospinal fluid (95), and also in many territories where klotho is not expressed.

**Figure 6:**
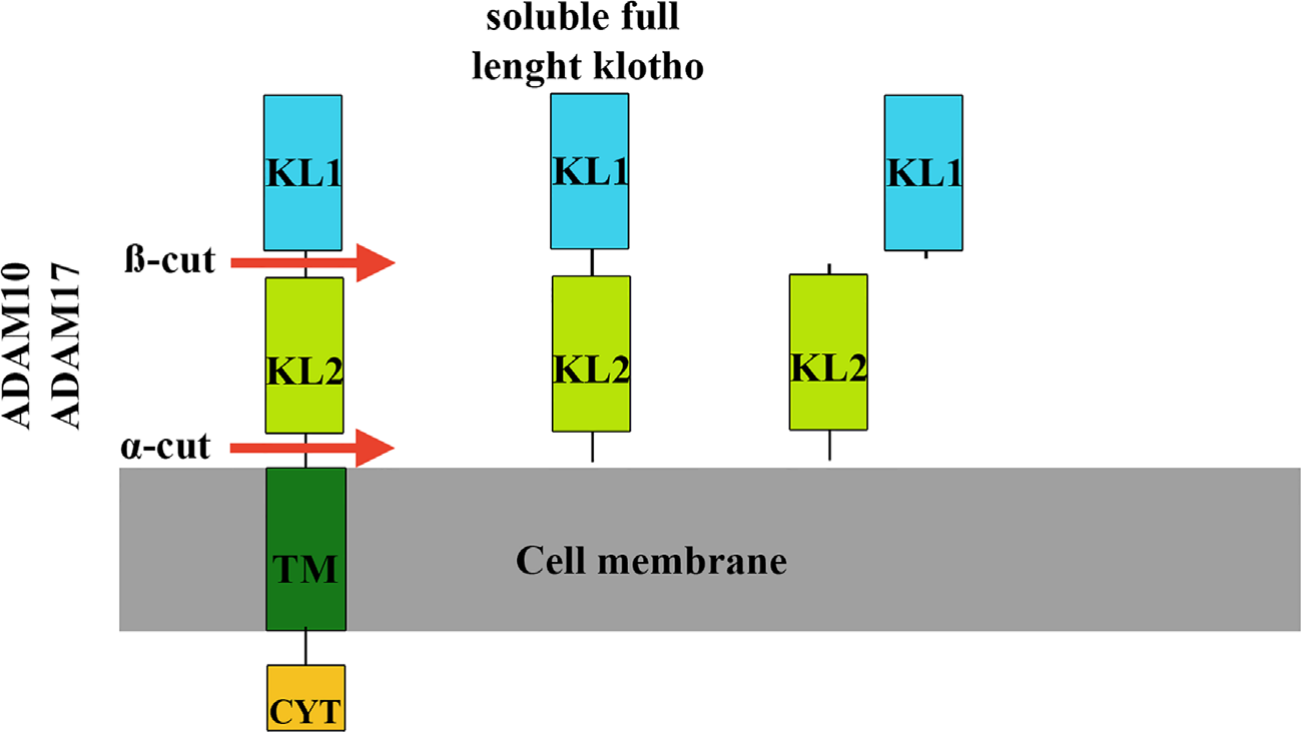
Klotho cleavage mechanisms. Klotho cleavage occurs in the extracellular domain of the klotho protein and is carried out by proteases (ADAM10 and ADAM17). In kidney cells, cleavage at a site directly above the cell membrane (α-cut) results in full-length circulating soluble klotho, which can act in many different organs and tissues. The other type of cleavage (ß-cut) occurs between the KL1 and KL2 domains that give rise to the KL2 and KL1 fragments. TM, membrane portion of klotho. CYT, intracellular portion of klotho.

The extracellular region of klotho contains two homologous domains, KL1 and KL2, which can be shed from the cell surface (96). Klotho cleavage occurs at a site directly above the plasma membrane (α-cut) or between the KL1 and KL2 domain (ß-cut), resulting in soluble full-length klotho or KL1 and KL2 fragments (97) (**Figure 6**). These different cleavages are carried out by proteases, including a disintegrin and metalloproteinase (ADAM10 and ADAM 17), mainly responsible for α-cut cleavage in kidney cells (97). After its release from the cell membrane, circulating soluble klotho exerts its biological effects on many different organs and tissues. There is no known receptor for soluble klotho, rather there is a coreceptor formed by shed klotho, fibroblast growth factor receptor (FGFR) and FGF23, indicating that shed klotho is an enzyme-dependent active scaffold protein (98). Thus, klotho is an essential cofactor for the binding of FGF23 to its receptor.

### Klotho Regulation and GH Secretion

**Figure 7:**
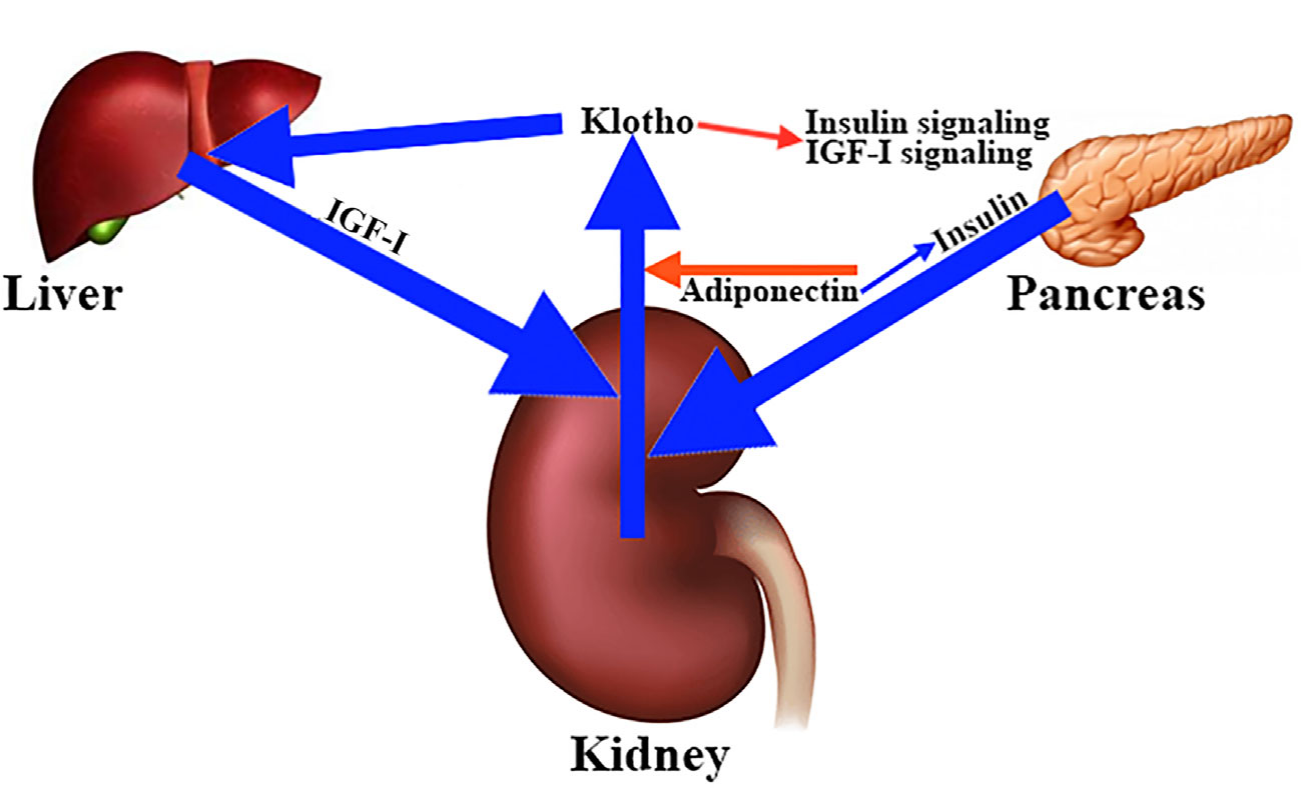
Main factors involved in the regulation of klotho secretion in the kidney. The kidneys are the main source of circulating klotho. Different hormones and factors involved in mineral homeostasis contribute to the regulation of klotho secretion. For example, adiponectin decreases renal klotho secretion (red arrow), while insulin stimulates cleavage and release of full-length soluble klotho (blue arrow), an effect that is sensitized by adiponectin (blue arrow). The same effect on klotho secretion is carried out by IGF-I (blue arrow), whose hepatic production appears to be stimulated by klotho (blue arrow). This is curious because klotho inhibits the insulin and IGF-I signaling pathways (red arrow).

To date, it has not been clearly established how klotho secretion is regulated, although the fact that the kidneys are the main source of klotho suggests that hormones and factors involved in mineral homeostasis play a role in this regulation. This is the case with adiponectin, a hormone that reduces renal secretion of klotho (99). Interestingly, adiponectin sensitizes insulin and insulin stimulates the cleavage and release of the extracellular domain of klotho (96). Similar to insulin, IGF-I appears to stimulate klotho secretion (22, 100), whereas klotho inhibits insulin/IGF-I signaling (101) (**Figure 7**), and activation of both hormone receptors (101). Interestingly, in mice, intraperitoneal injections of klotho for 4 weeks produced a significant increase in the levels of liver mRNA of IGF-I and its carrier protein IGFBP3 (102), which seems to contradict the inhibition that klotho exerts on the inhibitory effect of IGF-I in the pituitary secretion of GH. This and other studies indicate that klotho is a positive regulator of GH release. Perhaps this is the reason why *kl/kl* mice have hypotrophic somatotrophs and a reduced number of secretory granules (94).

**Figure 8:**
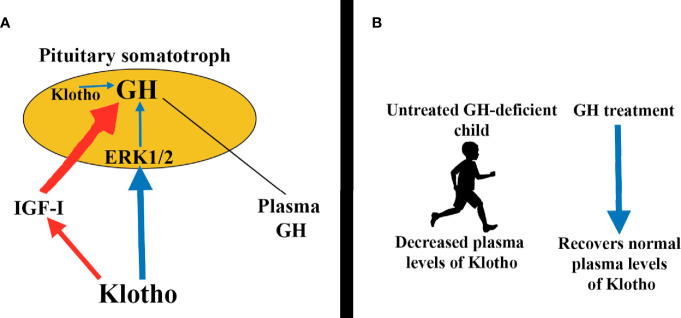
Klotho induces pituitary GH secretion. **(A)** Circulating klotho directly stimulates GH secretion by activating the ERK1/2 pathway (blue arrows). Indirectly, klotho also promotes GH secretion by inhibiting IGF-I and consequently its inhibitory effects on GH release (red arrows). Additionally, klotho is expressed in somatotrophs, perhaps to modulate auto/paracrine GH production (blue arrow). **(B)** Interestingly, plasma klotho levels are decreased in untreated GH-deficient children, but GH replacement therapy brings klotho to normal values.

The mechanisms by which klotho induces GH secretion involve the activation of the ERK1/2 pathway (**Figure 8A**), also demonstrated in GH3 cells (102); in these cultured cells cotreatment of klotho and bFGF further increased ERK1/2 phosphorylation, while inhibition of ERK1/2 activation leads to abolition of klotho-induced GH release in normal pituitaries (102). Klotho plasma levels are decreased in untreated GH-deficient children and adults, but increased during GH treatment (100) (**Figure 8B**). This was associated with an increase in plasma IGF-I levels dependent on the activation of Akt-mTOR pathway (100). However, in the case of klotho genetic deletions (*kl/kl* in mice) GH administration cannot lead to normal growth in these mice (103).

In acromegaly, increased levels of circulating klotho have been reported, returning to normal values shortly after surgery (104). This is most likely due to the fact that the elevated IGF-I levels that exist in this pathology lead to increased klotho secretion which, in turn, further increases GH secretion. Another possibility, not investigated, is that since klotho is also produced in the somatotrophs, perhaps to modulate auto/paracrine GH production, increased klotho in acromegaly could be a consequence of increased GH secretion; that is, klotho would be released from the pathological pituitary accompanying GH secretion.

Interestingly, a study in patients with pituitary adenomas showed that there was expression of klotho in both GH-secreting and non-GH-producing adenomas; this expression of klotho, proven by immunohistochemistry, was higher in non-GH-secreting adenomas, suggesting that non-GH-secreting pituitary cells are capable of producing klotho.

### Summary

Klotho is an anti-aging agent that is expressed primarily in the kidneys and the cerebral choroid plexus, but also in many other different tissues and organs, such as the gonads and the pituitary gland. Although it is a transmembrane protein, it can be found in the circulation, at the expense of proteolytic cleavage of its extracellular region. The soluble form of klotho thus generated is then capable of exerting multiple effects related to the mineral homeostasis of the organism, but also with physiological functions in different organs and tissues. Interestingly, there is no known receptor for klotho, rather there is a coreceptor formed by shed klotho, FGFR, and FGF23, which indicates that shed klotho is an enzyme-dependent active scaffold protein; therefore, klotho is a key cofactor for the binding of FGF23 to its receptor.

Among the multiple effects of klotho, we have to comment in relation to this review, the effect of klotho on pituitary GH secretion. Klotho induces GH secretion at the expense of ERK1/2 phosphorylation. Children and adults with untreated GH-deficiency show reduced plasma levels of klotho, but GH replacement therapies restore these low levels of klotho to normal values (**Figure 8B**). This does not occur when klotho is absent due to genetic mutations or deletions (*kl/kl* in mice, for example). In these situations, GH replacement therapy cannot lead to normal growth.

Insulin and IGF-I appear to stimulate the secretion of klotho, whereas klotho inhibits insulin/IGF-I signaling and the activation of both hormone receptors.

Klotho is expressed in pituitary adenomas and its expression is higher in non-GH-secreting adenomas than in GH-secreting adenomas, suggesting that non-GH-secreting pituitary cells are capable of producing klotho.

In summary, klotho is part of the complex world of regulating pituitary GH secretion, acting positively on it. The relationships between GH and klotho are summarized in **Table 3**.

**Table 3:** Effects of klotho on GH secretion and *vice versa*.

Level	Effect
**Anterior Pituitary**	+ Phosphorylation of ERK ½
	Paracrine induction of GH secretion
	Inhibits the inhibitory effect of IGF-I
**Plasma levels of klotho**	Decreased in untreated GH-deficient patients
	GH administration recovers normal values of klotho

+, Stimulates.

## Nesfatins

In 2006, a study described nesfatin-1 as a hypothalamic and brainstem peptide whose expression decreased during fasting, suggesting a role for this peptide in energy balance (105). Other studies using RT-PCR demonstrated that nesfatin-1 was expressed in various areas of the brain involved in metabolic regulation and eating behavior (105–108). Nesfatin-1 is also expressed in the adipose tissue and has been found in serum (108). The wide distribution of nesfatin-1 in the CNS indicates that this peptide also exerts endocrine and autonomic effects on energy expenditure (109); for example, nesfatin-1 has been found to be co-localized with neuroendocrine hormones, including GHRH or somatostatin, among others (109). Interestingly, nesfatin-1 has also been found to be produced in ghrelin-producing cells of the stomach (31), where it may be involved in the des-acyl ghrelin-induced inhibition of peripherally administered orexigenic ghrelin in free-fed rats (110).

In 2019, two DNA and calcium-binding peptides called nucleobindins (NUCB1 and NUCB2) were reported to be involved in many physiological processes as multifunctional regulators of cell biology, including activation of G protein signaling (111). These NUCBs can give rise to smaller peptides called nesfatin-1 (NESF) and nesfatin-1-like peptide (NLP) that share a 76.6% amino acid sequence identity with NESF (112). Although the full function of these peptides is not well understood, they suppress food intake and contribute to modulating energy homeostasis (106, 113–115), and they also produce endocrine effects, such as stimulating insulin secretion (112, 116) or regulating gonadal function (117, 118). From these data, it is feasible to assume that nesfatins are pleiotropic hormones that act through G-protein-coupled receptors (GPCR) (107, 119).

### Nesfatins Inhibit GH Synthesis and Secretion

In a very recent study, both NESF and NLP have been shown to inhibit GH synthesis and its ghrelin-induced release in mammalian somatotrophs (120). This study was carried out in cultured cell lines (GH3 and RC-4B/C) in which the authors demonstrated that both *nucb1* and *nucb2* mRNA expression existed, as well as their corresponding NUCB1 and NUCB2 proteins. NLP was found to be mainly located in the cytoplasm, while the distribution of NESF was more diffuse and was also found in the nucleus. Both NESF and NLP bind to the membrane of GH3 cells, suggesting the possibility of a GPCR-mediated action of NESF and NLP in these cells.

The expression of *gh* mRNA was down-regulated in these cells when incubated in the presence of low and high, but not medium, concentrations of these nesfatins. The same occurred with the expression of the pituitary-specific positive transcription factor 1 (*pit-1*), although in this case a significant down-regulation was only observed for NESF at 1 and 24 h of incubation, while NLP only led to a significant down-regulation of *pit-1* at 24 h. Consequently, GH protein levels decreased by an amount of approximately 31% at low and high concentrations of NESF after 1 and 6 h of incubation, while the decrease observed when incubating with NLP was slightly less (27%) in the same time periods.

**Figure 9:**
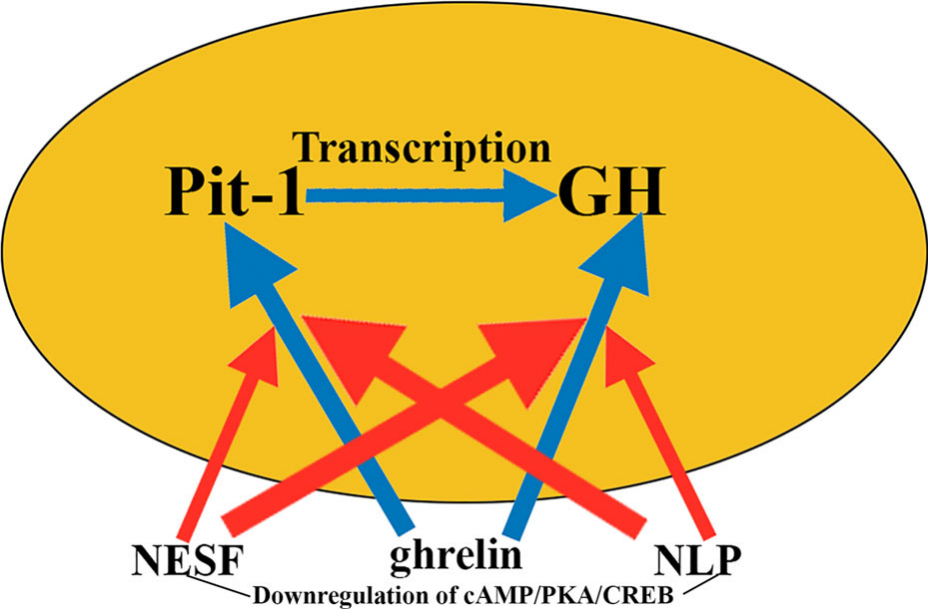
Nesfatins and GH secretion. Nesfatins are involved in suppressing food intake, although they also have many different endocrine effects. Therefore, it is logical that in GH cells cultured with ghrelin, which induces the expression of pit-1 and GH (blue arrows), the addition of nesfatins to the medium blocks, if not totally to a great extent, these effects of ghrelin on pit-1 and GH (red arrows), although its effects in intensity and time vary depending on the nesfatin used. The mechanism by which nesfatins induce these effects appears to be the downregulation of cAMP/PKA/CREB. NESF, nesfatin-1; NLP, nesfatin-1-like peptide.

Interestingly, the significant increase observed in *gh* and *pit-1* expression when GH3 cells were incubated in the presence of ghrelin was abrogated when these cells were pre-incubated or co-incubated with NESF or NLP. The signaling pathway responsible for these effects appears to be cAMP/PKA/CREB, which in this study was shown to be negatively regulated by both NESF and NLP (**Figure 9**).

Taken together, these results indicate that in mammalian somatotrophs, nesfatins play an inhibitory role on GH synthesis and secretion, although their physiological significance, for example in humans, has not yet been established.

### Summary

Although little is known about the role that nesfatins play in the human body, their wide distribution and the fact that they are circulating pleiotropic hormones indicates that they have to contribute to the modulation of many different physiological processes. For example, they have to act as counteracting hormones that inhibit the orexigenic effects of ghrelin, acting as signals that suppress food intake and modulate energy homeostasis. They also appear to act as neurohormones that modulate the function of many endocrine glands, such as the pancreas, gonads, and pituitary, acting through G-protein-coupled receptors.

In relation to its effects on the pituitary synthesis of GH and its secretion, the action of nesfatins is inhibitory. They reduce the expression of *pit-1*, and consequently that of the *gh* gene, by negatively regulating the cAMP/PKA/CREB signaling pathway and also block the stimulating effects of ghrelin on GH secretion in the pituitary. Given that these inhibitory effects have been demonstrated in cultured GH-producing cells, they appear to be paracrine and independent of any action on hypothalamic somatostatin release, although it would be interesting to analyze whether nesfatins play a role in hypothalamic control of the synthesis and secretion of pituitary GH exerted by SS-GHRH, or on the neurotransmitters involved in this hypothalamic regulation of GH. The fact that nesfatin-1 co-localizes with neuroendocrine hormones, such as GHRH or somatostatin, may support this possibility.

## Conclusions

Throughout this review, we have analyzed the effects of three peripheral hormonal factors, ghrelin, klotho, and nesfatins, on pituitary GH synthesis and secretion. Adding these factors to the world of GH regulation radically changes the classic concept of how pituitary GH is regulated by somatostatin and GHRH, and these neurohormones by hypothalamic adrenergic pathways (2). These three factors act basically at the pituitary level, although one of them, ghrelin, also performs its GH-secreting action facilitating the release of hypothalamic GHRH, perhaps inhibiting the hypothalamic release of somatostatin.

Interestingly, two of these factors, ghrelin and nesfatins, are involved in the regulation of energy homeostasis, although with opposite actions since, while ghrelin could be considered the hunger hormone, stimulating appetite, nesfatins would be the hormones of satiety, inhibiting orexigenic action of ghrelin. This may justify the fact that these hormones are involved in the control of the synthesis and secretion of GH, a hormone with important metabolic actions: hyperglycemic, lipolytic, and anabolic, which is stimulated by ghrelin and inhibited by nesfatins. Changes in the metabolic needs of the body at each moment of the day and throughout life would justify the secretion and actions of each of these hormones to optimize energy homeostasis at the expense of GH, among other factors. Interestingly, gastric ghrelin secretion decreases with age, as occurs with GH, while the pituitary ghrelin receptor remains present in the pituitary. It is very important that ghrelin has to be acylated by ghrelin O-acyl-transferase (GOAT), which binds a fatty acid side chain (C8) to serine 3 of ghrelin, forming acyl ghrelin, the active form that acts on its receptor, a G-protein-coupled receptor (GHSR-1a).

The other factor, klotho, is an anti-aging agent, the soluble circulating form of which directly induces GH secretion by activating ERK1/2 and inhibits the inhibitory effect that IGF-I exerts on GH. Klotho is also involved in the regulation of mineral homeostasis. Perhaps this is why the kidneys are the main source of this peptide. Klotho expression has also been found in the pituitary, where it perhaps induces a paracrine effect on GH synthesis and secretion. There are very important relationships between klotho and GH; untreated children and adults with GH-deficiency show reduced plasma levels of klotho, but GH treatment restores them to normal values (121). Deletions or mutations of the *klotho* gene affect GH production, but GH treatment cannot induce a normal growth in mice displaying these deletions (*kl/kl* mice).

In summary, these three factors must be added to the world of GH control. All of them act after being released from the peripheral level, directly on the pituitary gland (not yet proven for nesfatins) but they can also act on the GHRH-SS system and even paracrine in the pituitary. The broad spectrum of actions that these factors play in the body, along with their actions on GH, reinforces the idea that GH is more than just a growth hormone (11). The role of ghrelin and nesfatins in pituitary GH transcription is summarized in **Table 4**, along with the major GHRH-induced GH transcription factor Pit-1.

**Table 4:** Growth Hormone transcription factors.

FACTORS	Inducers	Inhibitors
Pit-1	+	
Ghrelin	+	
NESF and NLP		+
Klotho	?, +?	

+, Induces.

## Data Availability Statement

The raw data supporting the conclusions of this article will be made available by the authors, without undue reservation.

## Author Contributions

The author confirms being the sole contributor of this work and has approved it for publication.

## Funding

This review has been funded by Foundation Foltra, Teo, Spain, grant number Foltra 2020-06.

## Conflict of Interest

The author declares that the research was conducted in the absence of any commercial or financial relationships that could be construed as a potential conflict of interest.

## References

[1] Devesa J Lima L Lois N Fraga C Lechuga MJ Arce V. Reasons for the variability in growth hormone (GH) responses to GHRH challenge: the endogenous hypothalamic-somatotroph rhythm (HSR). Clin Endocrinol (Oxf) (1989) 30:367–77. 10.1111/j.1365-2265.1989.tb00434.x 2574645

[2] Devesa J Lima L Tresguerres JA. Neuroendocrine control of growth hormone secretion in humans. Trends Endocrinol Metab (1992) 3:175–83. 10.1016/1043-2760(92)90168-z 18407098

[3] Devesa J Arce V Lois N Tresguerres JA Lima L. Alpha 2-adrenergic agonism enhances the growth hormone (GH) response to GH-releasing hormone through an inhibition of hypothalamic somatostatin release in normal men. J Clin Endocrinol Metab (1990) 71:1581–8. 10.1210/jcem-71-6-1581 1977761

[4] Devesa J Díaz MJ Tresguerres JA Arce V Lima L. Evidence that alpha 2- adrenergic pathways play a major role in growth hormone (GH) neuroregulation: alpha 2-adrenergic agonism counteracts the inhibitory effect of muscarinic receptor blockade on the GH response to GH-releasing hormone, while alpha 2-adrenergic blockade diminishes the potentiating effect of increased cholinergic tone on such stimulation in normal men. J Clin Endocrinol Metab (1991) 73:251– 6. 10.1210/jcem-73-2-251 1677361

[5] Lima L Arce V Díaz MJ Tresguerres JA Devesa J. Clonidine pretreatment modifies the growth hormone secretory pattern induced by short-term continuous GRF infusion in normal men. Clin Endocrinol (Oxf) (1991) 35:129–35. 10.1111/j.1365-2265.1991.tb03510.x 1934527

[6] Lima L Arce V Tresguerres JA Devesa J. Studies on alpha 2-adrenergic Modulation of hypothalamic somatostatin secretion in rats. Life Sci (1993) 53:665–8. 10.1016/0024-3205(93)90277-a 8102470

[7] Lima L Arce V Tresguerres JA Devesa J. Clonidine potentiates the growth hormone (GH) response to GH-releasing hormone in norepinephrine synthesis-inhibited rats: evidence for an alpha 2-adrenergic control of hypothalamic release of somatostatin. Neuroendocrinology (1993) 57:1155–60. 10.1159/000126482 7901787

[8] Devesa J. Clonidine plus GHRH Administration for Diagnosing Growth Hormone Deficiency in Children. J Clin Mol Endocrinol (2017) 2:6–13. 10.21767/2572-5432.10042

[9] Alba-Roth J Muller OA Schopohl J von Werder K. Arginine stimulates growth hormone secretion by suppressing endogenous somatostatin secretion. J Clin Endocrinol Metab (1988) 67:1186–89. 10.1210/jcem-67-6-1186 2903866

[10] Ghigo E Bellone J Mazza E Imperiale E Procopio M Valente F. Arginine potentiates the GHRH- but not the pyridostigmine-induced GH secretion in normal short children. Further evidence for a somatostatin suppressing effect of arginine. Clin Endocrinol (1990) 32:763–67. 10.1111/j.1365-2265.1990.tb00923.x 1974484

[11] Devesa J Almengló C Devesa P. Multiple Effects of Growth Hormone in the Body: Is it Really the Hormone for Growth? Clin Med Insights Endocrinol Diabetes (2016) 9:47–71. 10.4137/CMED.S38201 27773998 PMC5063841

[12] Harvey S. Extrapituitary growth hormone. Endocrine (2010) 38:335–59. 10.1007/s12020-010-9403-8 20972718

[13] Pérez-Ibave DC Rodríguez-Sánchez IP Garza-Rodríguez ML Barrera-Saldaña HA. Extrapituitary growth hormone synthesis in humans. Growth Horm IGF Res (2014) 24:47–53. 10.1016/j.ghir.2014.01.005 24642386

[14] Pérez-Ibave DC Rodríguez-Sánchez IP Garza-Rodríguez ML Pérez-Maya AA Luna M Arámburo C. Expression of growth hormone gene in the baboon eye. Exp Eye Res (2018) 169:157–69. 10.1016/j.exer.2018.01.002 29407222

[15] Bowers CY Reynolds GA Durham D Barrera CM Pezzoli SS Thorner MO. Growth hormone (GH)-releasing peptide stimulates GH release in normal men and acts synergistically with GH-releasing hormone. J Clin Endocrinol Metab (1990) 70:975–82. 10.1210/jcem-70-4-975 2108187

[16] Guillemin R Brazeau P Böhlen P Esch F Wehremberg WB. Growth hormone-releasing factor from a human pancreatic tumor that caused acromegaly. Science (1982) 218:585–7. 10.1126/science.6812220 6812220

[17] Rivier J Spiess J Thorner M Vale W. Characterization of a growth hormone-releasing factor from a human pancreatic islet tumour. Nature (1982) 300:276–8. 10.1038/300276a0 6292724

[18] Howard AD Feighner SD Cully DF Arena JP Liberator PA Rosenblum CI. A receptor in pituitary and hypothalamus that functions in growth hormone release. Science (1996) 273:974–7. 10.1126/science.273.5277.974 8688086

[19] Kojima M Hosoda H Date Y Nakazato M Matsuo H Kangawa K. Ghrelin is a growth-hormone-releasing acylated peptide from stomach. Nature (1999) 402:656–60. 10.1038/45230 10604470

[20] Kojima M Kangawa K. Ghrelin: structure and function. Physiol Rev (2005) 85:495–522. 10.1152/physrev.00012.2004 15788704

[21] Rigamonti AE Pincelli AI Corrá B Viarengo R Bonomo SM Galimberti D. Plasma ghrelin concentrations in elderly subjects: comparison with anorexic and obese patients. J Endocrinol (2002) 175:R1–5. 10.1677/joe.0.175r001 12379512

[22] Caicedo D Díaz O Devesa P Devesa J. Growth Hormone (GH) and Cardiovascular System. Int J Mol Sci (2018) 19:290. 10.3390/ijms19010290 PMC5796235 29346331

[23] Baldanzi G Filigheddu N Cutrupi S Catapano F Bonisoni S Fubini A. Ghrelin and des-acyl ghrelin inhibit cell death in cardiomyocytes and endothelial cells through ERK1/2 and PI 3-kinase/AKT. J Cell Biol (2002) 159:1029–37. 10.1083/jcb.200207165 PMC2173981 12486113

[24] Muccioli G Tschöp M Papotti M Degenghi R Heiman M Ghigo E. Neuroendocrine and peripheral activities of ghrelin: implications in metabolism and obesity. Eur J Pharmacol (2002) 440:235–54. 10.1016/s0014-2999(02)01432-2 12007539

[25] Torsello A Ghé C Bresciani E Catapano F Ghigo E Deghenghi R. Short ghrelin peptides neither displace ghrelin binding in vitro nor stimulate GH release in vivo. Endocrinology (2002) 143:1968–71. 10.1210/endo.143.5.8894 11956180

[26] Hosoda H Kojima M Matsuo H Kangawa K. Ghrelin and des-acyl ghrelin: two major forms of rat ghrelin peptide in gastrointestinal tissue. Biochem Biophys Res Commun (2000) 279:909–13. 10.1006/bbrc.2000.4039 11162448

[27] Seim I Collet C Herington AC Chopin LK. Revised genomic structure of the human ghrelin gene and identification of novel exons, alternative splice variants and natural antisense transcripts. BMC Genomics (2007) 8:298. 10.1186/1471-2164-8-298 17727735 PMC2014779

[28] Date Y Kojima M Hosoda H Sawaguchi A Mondal MS Suganuma T. Ghrelin, a novel growth hormone-releasing acylated peptide, is synthesized in a distinct endocrine cell type in the gastrointestinal tracts of rats and humans. Endocrinology (2000) 141:4255–61. 10.1210/endo.141.11.7757 11089560

[29] Mizutani M Atsuchi K Asakawa A Matsuda N Fujimura M Inui A. Localization of acyl ghrelin-and des-acyl ghrelin-immunoreacive cells in the rat stomach and their responses to intragastric pH. Am J Physiol Gastrointest Liver Physiol (2009) 297:G974–80. 10.1152/ajpgi.00147.2009 20501445

[30] Ariyasu H Takaya K Tagami T Ogawa Y Hosoda K Akamizu T. Stomach is a major source of circulating ghrelin, and feeding state determines plasma ghrelin-like immunoreactivity levels in humans. J Clin Endocrinol Metab (2001) 86:4753–8. 10.1210/jcem.86.10.7885 11600536

[31] Stengel A Taché I. Ghrelin-a pleiotropic hormone secreted from endocrine x/a-like cells of the stomach. Front Neurosci (2012) 6:24. 10.3389/fnins.2012.00024 22355282 PMC3280431

[32] Cowley MA Smith RG Diano S Tschop M Pronchuk N Grove KL. The distribution and mechanism of action of ghrelin in the CNS demonstrates a novel hypothalamic circuit regulating energy homeostasis. Neuron (2003) 37:649–61. 10.1016/s0896-6273(03)00063-1 12597862

[33] Hou Z Miao Y Gao L Pan H Zhu S. Ghrelin-containing neuron in cerebral cortex and hypothalamus linked with the DVC of brainstem in rat. Regul Pept (2006) 134:126–31. 10.1016/j.regpep.2006.02.005 16600402

[34] Ueberberg B Unger N Saeger W Mann K Petersenn S. Expression of ghrelin and its receptor in human tissues. Horm Metab Res (2009) 41:814–21. 10.1055/s-0032-1314859 19670151

[35] Korbonits M Kojima M Kangawa K Grossman AB. Presence of ghrelin in normal and adenomatous human pituitary. Endocrine (2001) 14:101–4. 10.1385/ENDO:14:1:101 11322490

[36] Korbonits M Bustin SA Kojima M Jordan S Adams EF Lowe DG. The expression of the growth hormone secretagogue receptor ligand ghrelin in normal and abnormal human pituitary and other neuroendocrine tumors. J Clin Endocrinol Metab (2001) 86:881–7. 10.1210/jcem.86.2.7190 11158061

[37] Beiras-Fernández A Kreth S Weis F Ledderose C Pottinger T Diéguez C. Altered myocardial expression of ghrelin and its receptor (GHSR-1a) in patients with severe heart failure. Peptides (2010) 31:2222–8. 10.1016/j.peptides.2010.08.019 20804798

[38] Granata R Isgaard J Alloatti G Ghigo E. Cardiovascular actions of the ghrelin gene-derived peptides and growth hormone-releasing hormone. Exp Biol Med (Maywood) (2011) 236:505–14. 10.1258/ebm.2011.010365 21478211

[39] Lin B Liu Y Zhang W Zou W. Role of diet on intestinal metabolites and appetite control factors in SD rats. Exp Ther Med (2020) 20:2665–74. 10.3892/etm.2020.8993 PMC7401913 32765760

[40] Guan XM Yu H Palyha OC McKee KK Feighner SD Sirinathsinghji DJ. Distribution of mRNa encoding the growth hormone secretagogue receptor in brain and peripheral tissues. Brain Res Mol Brain Res (1997) 48:23–9. 10.1016/s0169-328x(97)00071-5 9379845

[41] Date Y Nakazato M Hashiguchi S Dezaki K Mondal MS Hosoda H. Ghrelin is present in pancreatic alpha-cells of humans and rats and stimulates insulin secretion. Diabetes (2002) 51:124–9. 10.2337/diabetes.51.1.124 11756331

[42] Gutierrez JA Solenberg PJ Perkins DR Willency JA Knierman MD Jin Z. Ghrelin octanoylation mediated by an orphan lipid transferase. Proc Natl Acad Sci USA (2008) 195:6320–5. 10.1073/pnas.0800708105 PMC2359796 18443287

[43] Yang J Brown MS Liang G Grishin NV Goldstein JL. Identification of the acyltransferase that octanoylates ghrelin, an appetite-stimulating peptide hormone. Cell (2008) 132:387–96. 10.1016/j.cell.2008.01.017 18267071

[44] Sakata I Nakamura K Yamazaki M Matsubara M Hayashi Y Kangawa K. Ghrelin-producing cells exist as two types of cells, closed- and opened-type cells, in the rat gastrointestinal tract. Peptides (2002) 23:531–6. 10.1016/s0196-9781(01)00633-7 11836003

[45] Armand M Hamosh M Mehta NR Angelus PA Philpott JR Henderson TR. Effect of human milk or formula on gastric function and fat digestion in the premature infant. Pediatr Res (1996) 40:429–37. 10.1203/00006450-199609000-00011 8865280

[46] Nishi Y Hiejima H Hosoda H Kaiya H Mori K Fukue Y. Ingested medium-chain fatty acids are directed utilized for the acyl modification of ghrelin. Endocrinology (2005) 146:2255–64. 10.1210/en.2004-0695 15677766

[47] Kirchner H Gutierrez JA Solenberg PT Pfluger PT Czyzyk TA Willency JA. GOAT links dietary lipids with the endocrine control of energy balance. Nat Med (2009) 15:741–5. 10.1038/nm.1997 PMC2789701 19503064

[48] Cummings DE Purnell JQ Frayo RS Schmidova K Wisse BE Weigle DS. A preprandial rise in plasma ghrelin suggests a role in meal initiation in humans. Diabetes (2001) 50:1714–9. 10.2337/diabetes.50.8.1714 11473029

[49] Lee H-M Wang G Englander EW Kojima M Greeley GH Jr. Ghrelin, a new gastrointestinal endocrine peptide that stimulates insulin secretion: enteric distribution, ontogeny, influence of endocrine, and dietary manipulations. Endocrinology (2002) 143:185–90. 10.1210/endo.143.1.8602 11751608

[50] Mundinger TO Cummings DE Taborsky GJ Jr. Direct stimulation of ghrelin secretion by sympathetic nerves. Endocrinology (2006) 147:2893–901. 10.1210/en.2005-1182 16527847

[51] Hosoda H Kangawa K. The autonomic nervous system regulates gastric ghrelin secretion in rats. Regul Pept (2008) 146:12–8. 10.1016/j.regpep.2007.07.005 17720259

[52] de la Cruz Dornonville C Norlén P Hakanson R. Secretion of ghelin from rat stomach ghrelin cells in response to local microinfusion of candidate messenger compounds: a microdialysis study. Regul Pept (2007) 143:118–26. 10.1016/j.regpep.2007.05.001 17573135

[53] Zhao T-J Sakata I Lin Li R Liang G Richardson JA Brown MS. Ghrelin secretion stimulated by ß1-adrenergic receptors in cultured ghrelinoma cells and in fasted mice. Proc Natl Acad Sci USA (2010) 107:15868–73. 10.1073/pnas.101111607 PMC2936616 20713709

[54] Sugino T Yamaura J Yamagishi M Kurose Y Kojima M Kangawa K. Involvement of cholinergic neurons in the regulation of the ghrelin secretory response to feeding in sheep. Biochem Biophys Res Commun (2003) 304:308–12. 10.1016/s0006-291x(03)00593-x 12711315

[55] Yin X Li Y Xu G An W Zhang W. Ghrelin fluctuation, what determines its production? Acta Biochim Biophys Sin (Shanghai) (2009) 41:188–97. 10.1093/abbs/gmp001 19280057

[56] Nonogaki K. Ghrelin and feedback systems. Vitam Horm (2008) 77:149–70. 10.1016/S0083-6729(06)77007-8 17983856

[57] Chabot F Caron A Laplante M St-Pierre DH. Interrelationships between ghrelin, insulin and glucose homeostasis: Physiological relevance. World J Diabetes (2014) 5:328–41. 10.4239/wjd.v5.i3.328 PMC4058737 24936254

[58] Broglio F Arvat E Benso A Gottero C Muccioli G Papotti M. Ghrelin, a natural GH secretagogue produced by the stomach, induces hyperglycemia and reduces insulin secretion in humans. J Clin Endocrinol Metab (2001) 86:5083–6. 10.1210/jcem.86.10.8098 11600590

[59] Polak P Hall MN. mTOR and the control of whole body metabolism. Curr Opin Cell Biol (2009) 21:209–18. 10.1016/j.ceb.2009.01.024 19261457

[60] Yoon M-S. The Role of Mammalian Target of Rapamycin (mTOR) in Insulin Signaling. Nutrients (2017) 9:1176. 10.3390/nu9111176 PMC5707648 29077002

[61] Xu G Li Y An W Zhao J Xiang X Ding L. Regulation of gastric hormones by systemic rapamycin. Peptides (2010) 31:2185–92. 10.1016/j.peptides.2010.08.018 PMC2995266 20804797

[62] Tong J Prigeon RL Davis HW Bidlingmaier M Tschöp MH D’Alessio D. Physiologic concentrations of exogenously infused ghrelin reduces insulin secretion without affecting insulin sensitivity in healthy humans. J Clin Endocrinol Metab (2013) 98:2536–43. 10.1210/jc.2012-4162 PMC3667259 23589527

[63] Arosio M Ronchi CL Gebbia C Cappiello V Beck-Peccoz P Peracchi M.Stimulatory effects of ghrelin on circulating somatostatin and pancreatic polypeptide levels. J Clin Endocrinol Metab (2003) 88:701–4. 10.1210/jc.2002-021161 12574202

[64] Azzam I Gilad S Limor R Stern N Greenman Y. Ghrelin stimulation by hypothalamic-pituitary-adrenal axis activation depends on increasing cortisol levels. Endocr Connect (2017) 6:847–55. 10.1530/EC-17-0212 PMC5682420 29038331

[65] Arafat MA Otto B Rochlitz H Tschop M Bahr V Mohlig M. Glucagon inhibits ghrelin secretion in humans. Eur J Endocrinol (2005) 153:397–402. 10.1530/eje.1.01981 16131602

[66] Kamegai J Tamura H Shimizu T Ishii S Sugihara H Oikawa S. Effects of insulin, leptin, and glucagon on ghrelin secretion from isolated perfused rat stomach. Regul Pept (2004) 119:77–81. 10.1016/j.reg.pep.2004.01.012 15093700

[67] Shimatsu A Kato Y Matsushita N Ohta H Kabayama Y Imura H. Glucagon-induced somatostatin release from perifused rat hypothalamus calcium dependency and effect of cysteamine treatment. Neurosci Lett (1983) 37:285–9. 10.1016/0304-3940(83)90445-7 6136940

[68] Gagnon J Anini Y. Glucagon stimulates ghrelin secretion through the activation of MAPK and EPAC and potentiates the effect of norepinephrine. Endocrinology (2013) 154:666–74. 10.1210/en.2012-1994 23307791

[69] Kalra SP Ueno N Kalra PS. Stimulation of appetite by ghrelin is regulated by leptin restraint: peripheral and central sites of action. J Nutr (2005) 135:1331–5. 10.1093/jn/135.5.1331 15867335

[70] Pöykkö SM Ukkola O Kauma H Kellokoski E Hörkkö S Kesäniemi YA. The negative association between plasma ghrelin and IGF-I is modified by obesity, insulin resistance and type 2 diabetes. Diabetologia (2005) 48:309–16. 10.1007/s00125-004-1635-9 15688209

[71] Whatmore AJ Hall CM Jones J Westwood M Clayton PE. Ghrelin concentrations in healthy children and adolescents. Clin Endocrinol (Oxf) (2003) 59:649–54. 10.1046/j.1365-2265.2003.01903.x 14616891

[72] Bellone S Rapa A Vivenza D Castellino N Petri A Bellone J. Circulating ghrelin levels as function of gender, pubertal status and adiposity in childhood. J Endocrinol Invest (2002) 25:RC13–5. 10.1007/BF03344026 12035950

[73] Bellone S Rapa A Vivenza D Vercellotti A Petri A Radetti G. Circulating ghrelin levels in newborns are not associated to gender, body weight and hormonal parameters but depend on the type of delivery. J Endocrinol Invest (2003) 26:RC9–RC11. 10.1007/BF03345172 12841532

[74] Stawerska R Smyczyriska J Hilczer M Lewinski A. Relationship between IGF-I Concentration and Metabolic Profile in Children with Growth Hormone Deficiency: The Influence of Children´s Nutritional State as the Ghrelin, Leptin, Adiponectin, and Resistin Serum Concentrations. Int J Endocrinol (2017) 2017:5713249. 10.1155/2017/571249 28596789 PMC5449754

[75] Shiiya T Nakazato M Mizuta M Date Y Mondal MS Tanaka M. Plasma ghrelin levels in lean and obese humans and the effect of glucose on ghrelin secretion. J Clin Endocrinol Metab (2002) 87:240–4. 10.1210/jcem.87.1.8129 11788653

[76] Nunez-Salces M Li H Feinle-Bisset C Young RL Page AJ. Nutrient-sensing components of the mouse stomach and the gastric ghrelin cell. Neurogastroenterol Motil (2020) 32:e13944. 10.1111/nmo.13944 32666613

[77] Dimaraki EV Jaffe CA Demott-Friberg R Russell-Aulet M Bowers CY Marbach P. Generation of growth hormone pulsatility in women: evidence against somatostatin withdrawal as pulse initiator. Am Physiol Endocrinol Metab (2001) 280:E489–95. 10.1152/ajpendo.2001.280.3.E489 11171604

[78] Dimaraki EV Jaffe CA Bowers CY Marbach P Barkan AL. Pulsatile and nocturnal growth hormone secretions in men do not require periodic declines of somatostatin. Am J Physiol Endocrinol Metab (2003) 285:E163–70. 10.1152/ajpendo.00334.2002 12670836

[79] Maheshwari HG Pezzoli SS Rahim A Shalet SM Thorner MO Baumann G. Pulsatile growth hormone secretion persists in genetic growth hormone-releasing hormone resistance. Am J Physiol Endocrinol Metab (2002) E943–51. 10.1152/ajpendo.00537.2001 11882517

[80] Mano-Otagiri A Nemoto T Sekino A Yamauchi N Shuto Y Sugihara H.Growth hormone-releasing hormone (GHRH) neurons in the arcuate nucleus (Arc) of the hypothalamus are decreased in transgenic rats whose expression of ghrelin receptor is attenuated: Evidence that ghrelin receptor is involved in the up-regulation of GHRH expression in the arc. Endocrinology (2006) 147:4093–103. 10.1210/en.2005-1619 16728494

[81] Zizzari P Halem H Taylor J Dong JZ Datta R Culler MD. Endogenous ghrelin regulates episodic growth hormone (GH) secretion by amplifying GH Pulse amplitude: evidence from antagonism of the GH secretagogue-R1a receptor. Endocrinology (2005) 146:3836–42. 10.1210/en.2005-0212 15919752

[82] Pantel J Legendre M Cabrol S Hilal L Hajaji Y Morisset S. Loss of constitutive activity of the growth hormone secretagogue receptor in familial short stature. J Clin Invest (2006) 116:760–8. 10.1172/JCI25303 PMC1386106 16511605

[83] Root A Root M. Clinical pharmacology of human growth hormone and its secretagogues. Curr Drug Targets Immune Endocr Metabol Disord (2002) 2:27–52. 10.2174/1568008024606293 12477295

[84] Tannenbaum GS Epelbaum J Bowers CY. Interrelationship between the novel peptide ghrelin and somatostatin/growth hormone-releasing hormone inregulation of pulsatile growth hormone secretion. Endocrinology (2003) 144:967–74. 10.1210/en.2002-220852 12586774

[85] Kamegai J Tamura H Shimizu T Ishii S Tatsuguchi A Sugihara H. The role of pituitary ghrelin in growth hormone (GH) secretion: GH-releasing hormone-dependent regulation of pituitary ghrelin gene expression and peptide content. Endocrinology (2004) 145:3731–8. 10.1210/en.2003-1424 15087428

[86] Rodríguez-Pacheco F Luque RM Tena-Sempere M Malagón MM Castaño JP. Ghrelin induces growth hormone secretion via a nitric oxide/cGMP signalling pathway. J Neuroendocrinol (2008) 20:406–12. 10.1111/j.1365-2826-2008-01645.x 18208548

[87] Magdaleno-Méndez A Domínguez B Rodríguez-Andrade A Barrientos-Morales M CervantesAcosta P Hernández-Beltrán A. Ghrelin increases growth hormone production and functional expression of NaV1.1 and NaV1.2 channels in pituitary somatotropes. Endocrine (2015) 48:929–36. 10.1007/s120200-014-0392-x 25151402

[88] Vázquez MJ Novelle MG Rodríguez-Pacheco F Lage R Varela L López M. AMPK-Dependent Mechanisms but Not Hypothalamic Lipid Signaling Mediates GH-Secretory Responses to GHRH and Ghrelin. Cells (2020) 9:1940. 10.3390/cells9091940 PMC7564832 32839401

[89] Benso A Gramaglia E Prodam F Riganti F Ramella Gigiardi V Lucatello B. Beta-adrenergic agonism does not impair the GH response to acylated ghrelin in humans. Clin Endocrinol (Oxf) (2009) 71:234–36. 10.1111/j.1365-2265.2008.03488.x 19067721

[90] Nass R Johannsson G Christiansen JS Kopchick JJ Thorner MO. The aging population—is there a role for endocrine interventions? Growth Horm IGF Res (2009) 19:89–100. 10.1016/j.ghir.2008.09.002 18977675

[91] Nass R Liu J Pezzoli S Oliveri M Gaylinn B Thorner M. 24-h Mean acyl-ghrelin levels are decreased in older adults. In: 4th International Congress of the GRS and the IGF Society. Italy: Springer Link (2008). p. p.51.

[92] Sun Y Garcia JM Smith RG. Ghrelin and growth hormone secretagogue receptor expression in mice during aging. Endocrinology (2007) 148:1323–9. 10.1210/en.2006-0782 17158206

[93] Broglio F Benso A Castiglioni C Gottero C Prodam F Destefanis S. The endocrine response to ghrelin as a function of gender in humans in young and elderly subjects. J Clin Endocrinol Metab (2003) 88:1537–42. 10.1210/jc.2002-021504 12679436

[94] Kuro-O M Matsumura Y Aizawa H Kawaguchi H Suga T Utsugi T. Mutation of the mouse klotho gene leads to a síndrome resembling ageing. Nature (1997) 390:45–51. 10.1038/36285 9363890

[95] Imura A Iwano A Tohyama O Tsuji Y Nozaki K Hashimoto N. Secreted Klotho protein in sera and CSF: implication for post-translational cleavage in release of Klotho protein from cell membrane. FEBS Lett (2004) 5651:143–7. 10.1016/j.febslet.2004.03.090 15135068

[96] Chen CD Podvin S Gillespie E Leeman SE Abraham CR. Insulin stimulates the cleavage of and release of the extracellular domain of Klotho by ADAM10 and ADAM 17. Proc Natl Acad Sci USA (2007) 104:19796–801. 10.1073/pnas.0709805104 PMC2148378 18056631

[97] Van Loon EPM Pulskens WP van der Hagen EAE Lavrijsen M Vervloet MC van Goor H. Shedding of Klotho by ADAMs in the kidney. Am J Physiol Renal Physiol (2015) 309:F359–68. 10.1152/ajprenal.00240.2014 26155844

[98] Chen G Liu Y Goetz R Fu L Jayaraman S Hu MC. α-Klotho is a non-enzymatic molecular scaffold for FGF23 hormone signaling. Nature (2018) 553:461–6. 10.1038/nature25451 PMC6007875 29342138

[99] Rutkowski JR Pastor J Sun K Park AC Bobulescu IA Chen CT. Adiponectin alters renal calcium and phosphate excretion through regulation of klotho expression. Kidney Int (2017) 91:324–37. 10.1016/j.kint.2016.09.016 PMC5237401 27914707

[100] Rubinek T Shahmoon S Shabtay-Orbach A Ben Ami M Levy-Shraga Y Mazor-Aronovitch K. Klotho response to treatment with growth hormone and the role of IGF-I as a mediator. Metabolism (2016) 65:1597–604. 10.1016/j.metabol.2016.08.004 27733247

[101] Kurosu H Yamamoto M Clark JD Pastor JV Nandi A Gurnani P. Suppression of aging in mice by the hormone Klotho. Science (2005) 309:1829–33. 10.1126/science.1112766 PMC2536606 16123266

[102] Shahmoon S Rubinfeld H Wolf I Cohen ZR Hadami M Shimon H. The aging suppressor klotho: a potential regulator of growth hormone secretion. Am J Physiol Endocrinol Metab (2014) 307:E3326–E334. 10.1152/ajpendo.00090.2014 24939736

[103] Kashimada K Yamashita T Tsuji K Nifuji A Mizutani S Nabeshima Y. Defects in growth and bone metabolism in klotho mutant mice are resistant to GH treatment. J Endocrinol (2002) 174:403–10. 10.1677/joe.0.1740403 12208660

[104] Neidert MC Sze L Zwimpfer C Sarnthein J Seifert B Frei K. Soluble α Klotho: a novel serum biomarker for the activity of GH-producing pituitary adenomas. Eur J Endocrinol (2013) 168:575–83. 10.1530/EJE-12-1045 23360820

[105] Cowley MA Grove KL. To be or NUCB2, is nesfatin the answer? Cell Metab (2006) 4:421–2. 10.1016/j.cmet.2006.11.001 17141625

[106] Oh-I S Shimizu H Satoh T Okada S Adachi S Inoue K. Identification of nesfatin-1 as a satiety molecule in the hypothalamus. Nature (2006) 443:709–12. 10.1038/nature05162 17036007

[107] Brailoiu GC Dun SL Brailoiu E Inan S Yang J Chang JK. Nesfatin-1:distribution and interaction with a G protein coupled receptor in the rat brain. Endocrinology (2007) 148:5088–94. 10.1210/en.2007-0701 17627999

[108] Shimizu H Ohsaki A Oh-I S Okada S Mori M. A new anorexigenic protein, nesfatin-1. Peptides (2009) 30:995–8. 10.1016/j.peptides.2009.01.002 19452636

[109] Foo KS Brismar H Broberger C. Distribution and neuropeptide coexistence of nucleobinding- 2 mRNA/nesfatin-like immunoreactivity in the rat CNS. Neuroscience (2008) 156:563–79. 10.1016/j.neuroscience.2008.07.054 18761059

[110] Inhoff T Mönnikes H Noetzel S Stengel A Goebel M Dinh QT. Desacyl ghrelin inhibits the orexigenic effect of peripherally injected ghrelin in rats. Peptides (2008) 29:2159–168. 10.1016/j.peptides.2008.09.014 PMC2586396 18938204

[111] Leung AKW Ramesh N Vogel C Unniapan S. Nucleobindins and encoded peptides: from cell signaling to physiology. Adv Protein Chem Struct Biol (2019) 116:91–133. 10.1016/bs.apcsb.2019.02.001 31036300

[112] Ramesh N Mohan H Unniappan S. Nucleobindin-1 encodes a nesfatin-1-like peptide that stimulates insulin secretion. Gen Comp Endocrinol (2015) 216:182–9. 10.1016/j.ygcen.2015.04.011 25907657

[113] Schalla MA Stengel A. Current Understanding of the Role of Nesfatin-1. J Endocr Soc (2018) 2:1188–2016. 10.1210/js.2018-00246 30302423 PMC6169466

[114] Mortazavi S González R Ceddia R Unniappan S. Long-term infusion of nesfatin-1 causes a sustained regulation of whole-body energy homeostasis in male Fischer 344 rats. Front Cell Dev Biol (2015) 3:22. 10.3389/fcell.2015.00022 25905102 PMC4389570

[115] Gawli K Ramesh N Unniappan S. Nesfatin-1-like peptide is a novel metabolic factor that suppresses feeding, and regulates whole-body energy homeostasis in male Wistar rats. PloS One (2017) 12:e0178329. 10.1371/journal.pone.0178329 28542568 PMC5444818

[116] Prinz P Stengel A. Nesfatin-1: current status as a peripheral hormone and future prospects. Curr Opin Pharmacol (2016) 31:19–24. 10.1016/j.coph.2016.08.011 27589696

[117] Kim J Yang H. Nesfatin-1 as a new potent regulator in reproductive system. Dev Reprod (2012) 16:253–64. 10.12717/DR.2012.16.4.253 PMC4282246 25949098

[118] Gao X Zhang K Song M Li X Luo L Tian Y. Role of Nesfatin-1 in the Reproductive Axis of Male Rat. Sci Rep (2016) 6:32877. 10.1038/srep32877 27599613 PMC5013388

[119] Ishida E Hashimoto K Shimizu H Okada S Satoh T Kato I. Nesfatin-1 induces the phosphorylation levels of cAMP response element-binding protein for intracellular signaling in a neural cell line. PloS One (2012) 7:e50918. 10.1371/journal.pone.0050918 23236405 PMC3516497

[120] Vélez E Unniappan S. Nesfatin-1 and nesfatin-1-like peptide suppress growth hormone synthesis via the AC/PKA/CREB pathway in mammalian somatotrophs. Sci Rep (2020) 10:16686. 10.1038/s41598-020-73840-4 33028951 PMC7541516

[121] Sato T Komaba H Nagatani T Watanabe T Kishida Y Fukagawa M. The pituitary is a candidate organ that modulates circulating Klotho levels. J Endocr Soc (2018) 3:52–61. 10.1210/js.2018-00223 30697600 PMC6344344

